# Transcranial magnetic stimulation (TMS) therapy for autism: an international consensus conference held in conjunction with the international meeting for autism research on May 13th and 14th, 2014

**DOI:** 10.3389/fnhum.2014.01034

**Published:** 2015-01-06

**Authors:** Lindsay M. Oberman, Peter G. Enticott, Manuel F. Casanova, Alexander Rotenberg, Alvaro Pascual-Leone, James T. McCracken

**Affiliations:** ^1^Neuroplasticity and Autism Spectrum Disorder Program, Department of Psychiatry and Human Behavior, E.P. Bradley Hospital and Warren Alpert Medical School, Brown UniversityProvidence, RI, USA; ^2^Cognitive Neuroscience Unit, School of Psychology, Deakin UniversityBurwood, VIC, Australia; ^3^Department of Psychiatry and Behavioral Science, University of LouisvilleLouisville, KY, USA; ^4^Neuromodulation Program, Department of Neurology, Boston Children's Hospital and Harvard Medical SchoolBoston, MA, USA; ^5^Berenson-Allen Center for Noninvasive Brain Stimulation, Department of Neurology, Beth Israel Deaconess Medical Center and Harvard Medical SchoolBoston, MA, USA; ^6^Department of Psychiatry and Biobehavioral Sciences, David Geffen School of Medicine at University of California, Los AngelesLos Angeles, CA, USA

**Keywords:** Autism Spectrum Disorder, transcranial magnetic stimulation, pathophysiology, therapy, consensus

The Centers for Disease Control and Prevention currently estimate the prevalence of Autism Spectrum Disorder (ASD) in the U.S. at 1:68 children (Baio, 2014). Despite decades of research across multiple levels of analysis, we currently lack a reliable biomarker that may facilitate diagnosis, illuminate pathophysiology, or guide treatment. The development of novel treatment strategies for ASD will require efforts for better clinical characterization, identification of more homogeneous subgroups for studies, and improved understanding of underlying pathophysiology. There is growing support for early intensive interventions in this population (Reichow, 2012). Pharmacological treatments have been shown to be effective in treating some of the common secondary and comorbid features of ASD (Hampson et al., 2012), but there is currently no pharmacotherapy conclusively shown to improve the core symptoms (Oberman, 2012).

Recently a number of investigators have begun to explore the use of transcranial magnetic stimulation (TMS) as a tool to characterize ASD pathophysiology, and to test its therapeutic potential. TMS is a safe and well-tolerated method for non-invasive focal cortical stimulation where small intracranial electrical currents are generated by a rapidly fluctuating extracranial magnetic field. In an effort to share recent progress in the use of TMS in ASD, promote collaboration across laboratories, and establish consensus on parameters that may be useful for the study of pathophysiology and the potential treatment of ASD, leading experts in the field gathered in Atlanta, GA on May 13th and 14th 2014 for the “Transcranial Magnetic Stimulation (TMS) Therapy for Autism Consensus Conference” organized and supported by the Clearly Present Foundation with additional support from Neuronetics, Inc. and Autism Speaks.

Alvaro Pascual-Leone began the conference by discussing the basic mechanisms and safety of TMS in clinical populations. TMS can be applied in single pulses to investigate corticospinal excitability, pairs of pulses to study intracortical inhibition and facilitation, and repeated trains of TMS (rTMS) to both to study and therapeutically modulate excitability and plasticity in a number of neurological and psychiatric conditions (Kobayashi and Pascual-Leone, 2003). The effects of rTMS can be expected to differ considerably by virtue of varying parameters of stimulation and knowledge of underlying symptom pathophysiology. TMS is considered quite safe if applied within current safety guidelines; however, it does pose some risk for adverse side-effects (Rossi et al., 2009). Though relatively few patients with ASD have participated in TMS protocols, the frequency and quality of side-effects shown thus far approximates that seen in the general population (Oberman et al., 2013). As with any other condition, factors including medications and medical history need to be assessed when determining risk for an individual. There are currently no identified ASD-specific risk factors for TMS-induced adverse effects. Even though ASD can be associated with an increased risk for seizures, in TMS studies to date, there is no evidence of increased epileptogenic risk in ASD when safety guidelines and recommendations are followed.

Manuel Casanova then provided a targeted review of the literature on the pathophysiology of ASD. Postmortem studies have shown evidence of abnormalities of neuronal migration in the brains of individuals with ASD (Bailey et al., 1998), which include displaced neurons manifesting as focal cortical dysplasias in a majority of individuals with ASD (Casanova et al., 2013). Morphometric analysis of cells within the malformed cortex has suggested a reduced number of interneurons (Casanova et al., 2013). This is consistent with previous reports of abnormalities in ASD within the peripheral cortical minicolumn neuropil space, the compartment where most inhibitory cells are located (Casanova et al., 2002). Both EEG and vibrotactile studies corroborate a deficit of cortical lateral inhibition (Keita et al., 2011; Puts et al., 2014). He proposed that this deficit could account for the seizures and sensory abnormalities often reported in ASD.

Lindsay Oberman discussed the use of TMS as an investigative device to study cortical excitability and plasticity in ASD. These studies show that a number of basic mechanisms and circuits are atypical while other measures appear to be normal (see Oberman et al., 2013). Specifically, motor thresholds and baseline motor-cortical excitability measures appear to be normal. There is heterogeneity in the response to paired-pulse paradigms with impaired inhibition in some individuals, typical response in others, and paradoxical facilitation in another subgroup. Studies exploring corticospinal plasticity mechanisms, using two different rTMS protocols [theta burst stimulation (TBS) and paired associative stimulation (PAS)], have shown abnormalities. However, the direction of the abnormality is unclear with TBS studies showing enhanced response (Oberman et al., 2012) and PAS showing reduced response (Jung et al., 2013). There are a number of open questions related to the use of TMS as an investigative device in ASD including developmental effects, effects related to intellectual disability and functioning, and what underlying mechanisms are driving the observed heterogeneity in the population.

Peter Enticott discussed the efficacy of rTMS as a therapeutic intervention in ASD. A number of studies using low-frequency rTMS in an effort to enhance cortical inhibitory tone in dorsolateral prefrontal cortex have resulted in improvements in EEG indices of attention, information processing, and error monitoring as well as behavioral improvements in repetitive behaviors and irritability (Sokhadze et al., 2014). Low-frequency stimulation to left pars triangularis resulted in improved object naming in a single session study (Fecteau et al., 2011). High-frequency stimulation, designed to enhance excitability, has suggested improvements in self-reported social relating and social anxiety following medial prefrontal cortex stimulation (Enticott et al., 2014) and significant improvements in eye-hand coordination following premotor stimulation (Panerai et al., 2013). Although an emerging literature, these studies collectively provide support for the potential efficacy of rTMS in ASD (Oberman et al., 2013). However, the small study samples, lack of blind assessments, and limited use of control or comparison conditions limit the interpretation of these early investigations.

James McCracken concluded the conference by discussing key factors to consider when designing clinical trials for ASD. These factors included identification of valid and reliable endpoints, incorporation of blind assessments, need for credible control conditions, establishment of effective stimulation parameters, need to relate changes in electrophysiologic endpoints to functional change, and identification of biomarkers that can be used to reduce the heterogeneity of the sample and stratify participants to treatment strategies that are best matched to their underlying pathophysiology. To this end, those present discussed the utility of developing functional imaging and TMS indices as potential standardized biomarkers and the need for larger, multisite trials to establish validity of these measures across development and levels of functioning and reliability of these measures across centers.

At the conclusion of the conference, there was enthusiasm for the potential use of TMS in ASD. Further work is necessary to achieve consensus on the key factors discussed by Dr. McCracken, but the expertise and commitment is present in the research and clinical community to work toward the end goal of designing and implementing large-scale, double blind, multisite clinical trials of rTMS for ASD in the near future. Those present committed to collaborate across laboratories to establish mutually agreed upon protocols and to meet again within 1 year.

## Author contributions

Lindsay M. Oberman, Peter G. Enticott, Manuel F. Casanova, Alvaro Pascual-Leone, and James T. McCracken contributed to the organization of the conference, were invited speakers to the conference, and lead the discussion during the conference. Alexander Rotenberg contributed to the organization of the conference, and the discussion during the conference. All authors contributed to the writing of the manuscript.

### Conflict of interest statement

Alvaro Pascual-Leone serves on the scientific advisory boards for Nexstim, Neuronix, Starlab Neuroscience, Axilum Robotics, Magstim, Neuroelectrics, and Neosync; and is listed as an inventor on several issued and pending patents on the real-time integration of transcranial magnetic stimulation (TMS) with electroencephalography (EEG) and magnetic resonance imaging (MRI). Alexander Rotenberg is listed as an inventor on a patent for apparatus and method of use of TMS in epilepsy. He is a co-founder of Neuromotion Inc. This conference was supported by the Clearly Present Foundation, Autism Speaks, and Neuronetics, Inc. The authors declare that the research was conducted in the absence of any commercial or financial relationships that could be construed as a potential conflict of interest.
